# Artificial intelligence for phase recognition in complex laparoscopic cholecystectomy

**DOI:** 10.1007/s00464-022-09405-5

**Published:** 2022-08-08

**Authors:** Tomer Golany, Amit Aides, Daniel Freedman, Nadav Rabani, Yun Liu, Ehud Rivlin, Greg S. Corrado, Yossi Matias, Wisam Khoury, Hanoch Kashtan, Petachia Reissman

**Affiliations:** 1 Verily Life Sciences, Tel Aviv, Israel; 2 Google Health, Tel Aviv, Israel; 3 Google Research, Tel Aviv, Israel; 4 Department of Surgery, Rappaport Faculty of Medicine, Carmel Medical Center, Technion, Haifa, Israel; 5 Department of Surgery, Rabin Medical Center, The Sackler School of Medicine, Tel-Aviv University, Petah Tikva, Israel; 6 Department of Surgery, The Hebrew University School of Medicine, Sharee Zedek Medical Center, Jerusalem, Israel; 7 Digestive Disease Institute, Shaare-Zedek Medical Center, The Hebrew University School of Medicine, P.O. Box 3235, 91031 Jerusalem, Israel

**Keywords:** Artificial Intelligence, Cholecystectomy, Computer Vision

## Abstract

**Background:**

The potential role and benefits of AI in surgery has yet to be determined. This study is a first step in developing an AI system for minimizing adverse events and improving patient’s safety. We developed an Artificial Intelligence (AI) algorithm and evaluated its performance in recognizing surgical phases of laparoscopic cholecystectomy (LC) videos spanning a range of complexities.

**Methods:**

A set of 371 LC videos with various complexity levels and containing adverse events was collected from five hospitals. Two expert surgeons segmented each video into 10 phases including Calot’s triangle dissection and clipping and cutting. For each video, adverse events were also annotated when present (major bleeding; gallbladder perforation; major bile leakage; and incidental finding) and complexity level (on a scale of 1–5) was also recorded. The dataset was then split in an 80:20 ratio (294 and 77 videos), stratified by complexity, hospital, and adverse events to train and test the AI model, respectively. The AI-surgeon agreement was then compared to the agreement between surgeons.

**Results:**

The mean accuracy of the AI model for surgical phase recognition was 89% [95% CI 87.1%, 90.6%], comparable to the mean inter-annotator agreement of 90% [95% CI 89.4%, 90.5%]. The model’s accuracy was inversely associated with procedure complexity, decreasing from 92% (complexity level 1) to 88% (complexity level 3) to 81% (complexity level 5).

**Conclusion:**

The AI model successfully identified surgical phases in both simple and complex LC procedures. Further validation and system training is warranted to evaluate its potential applications such as to increase patient safety during surgery.

**Supplementary Information:**

The online version contains supplementary material available at 10.1007/s00464-022-09405-5.

Computer vision based artificial intelligence (AI) systems have been successfully used for various purposes, though their utility for analysis or to aid in safety [1, 2] during surgical procedures is still under early evaluation. These investigations are important; for example during laparoscopic cholecystectomy (LC), adverse events such as bile duct injury, bile leakage, bleeding, and bowel injury events are possible, at a rate of 1.5% [3–9].

An AI system which can recognize surgical phases, may be used for many important tasks like quality measures, adverse events recording and analysis, education, statistics, surgical performance evaluation and more. Currently, these tasks are performed manually in a time consuming fashion by expert surgeons. Use of the system during surgery would further enable real-time monitoring and assisted decision making, which may increase safety and improve patient outcomes. For example, a real-time assistive system could alert the surgeon to an incorrect plane of dissection, a wrong maneuver, or an upcoming complication. Such a system might also be used as a context-aware decision support system by providing early warnings in case of misorientation or other unexpected events. As a specific example in LC, achieving the Critical View of Safety (CVS) is the recommended strategy for minimizing the risk of Bile Duct Injury (BDI) [10, 11]. A system that can detect and verify that CVS has been achieved is potentially quite valuable. The system can also optimize operating room (OR) utilization and staff scheduling, and provide administrative assistance by analyzing the progress of an operation and more accurately predicting the time required for procedure completion.

Computer Vision (CV) algorithms have recently shown success in recognizing surgical phases in LC procedures without adverse events [12, 13], and have displayed promising results in the ability of Artificial Intelligence (AI) to verify CVS during LC [14]. While these works [12, 13] have focused primarily on LC procedures without complications and adverse events, our hypothesis was that CV could also recognize surgical phases in more complex LC procedures. We therefore developed an AI system to recognize the major phases of both straightforward and complicated LC procedures with potentially higher morbidity rates.

## Methods

### Dataset

We constructed a dataset of 448 cholecystectomy videos, which includes 368 videos which were collected from four hospitals in Israel, and 80 videos from the publicly available Cholec80 dataset [12] collected from a hospital in France. The videos were recorded between November 1, 2010 and October 1, 2020. Eligibility criteria were laparoscopic cholecystectomy for biliary colic or acute and chronic cholecystitis, as well as patients 18 years of age or older. After excluding videos that could not be annotated consistently by surgeons (see the Annotation section), 371 videos remained and were used for this work. The dataset was split in an 80:20 ratio, respectively, for training and testing the AI model, with the splits stratified by surgical complexity, institution, and adverse events during surgery (see the Annotation section below). The splitting was performed on a per-case rather than a per-frame level. That is, frames from a video in the training set did not appear in the test set.

IRB approval was granted prior to commencing the study.

### Annotation of surgical phases, adverse events, and level of surgical complexity

#### Surgical phases and adverse events annotation

All datasets (including the publicly available Cholec80) were annotated. The relevant phases and annotation process was determined via consensus of a group of three experienced senior surgeons (years of experience: 35, 34, and 20), who were distinct from the surgeons who annotated the videos (described below). Each video was annotated according to the following phases: (1) trocar insertion, (2) preparation, (3) Calot triangle dissection, (4) clipping and cutting, (5) gallbladder dissection, (6) gallbladder packaging, (7) cleaning and hemostasis, and (8) gallbladder extraction. Additionally, two special phases were used in annotation. First, segments in which the camera was not placed inside the body were annotated as “out of body”. Second, segments in which the camera was not focused on tools and no surgical action was being performed were annotated as “idle”.

To analyze the ability of the AI model to recognize the major surgical phases in videos of abnormal or challenging LC procedures, a set of important adverse events were also identified by the expert surgeons. The experts agreed on the following list of adverse events, which were therefore annotated (where present): (1) major bleeding, (2) gallbladder perforation, (3) major bile leakage, and (4) incidental finding.

#### Surgical complexity annotation

In addition to annotating the phases and adverse events described above, annotations were also collected for the complexity level of each procedure. The complexity level was scored on a scale of 1–5 based on intraoperative parameters. The factors to determine the complexity level included state of the gallbladder (based on the Parkland Grading Scale for grading still images of Cholecystitis [15, 16]), presence of intra-abdominal adhesions, normality of anatomy, duct closure device utilized, performance of intraoperative cholangiography, partial or open cholecystectomy requirements and intraoperative adverse events. A detailed mapping between each procedure to its complexity level is described in Table 1 in the Supplement. The annotations of complexity levels and complications were used for assessing the AI model’s ability to accurately recognize the surgical phases in complex LC procedures.

#### Critical view of safety annotation

The last annotation task was the annotation of the Critical View of Safety (CVS), if achieved, during the Calot triangle dissection phase. We followed the three criteria defined by SAGES to annotate achievement of CVS: (1) the hepatocystic triangle is cleared of fat and fibrous tissue; (2) the lower third of the gallbladder is separated from the liver to expose the cystic plate; and (3) exactly two structures are seen entering the gallbladder.

#### Annotation quality

The annotations were performed by 13 surgeons with at least 4 years of experience (median: 7, range: 4–15) in general surgery. Annotator training included understanding the definition of each phase and adverse event; learning how to indicate the start and end of each phase; and becoming familiar with the annotation software. To validate the quality of the annotations, each video was annotated by two annotators, and the inter-rater agreement score between them was calculated. The inter-rater agreement score is defined as the number of frames annotated with the same phase label by the two annotators, divided by the total number of annotated frames in the video. Videos with an agreement score below 80% (n = 77) were excluded to arrive at the final set of 371 videos in the dataset (Table 1 and eTable 2 in the Supplement). The videos excluded from the main analysis are analyzed in eFigure 1 in the Supplement.

**Table 1:** General characteristics of the collected dataset

Variable		Training	Testing
No. of sites		5	5
No. of videos		294	77
No. frames (1 FPS)		586,453	107,819
Complexity level	1 (%)	58 (20%)	21 (27%)
	2 (%)	125 (43%)	38 (49%)
	3 (%)	43 (15%)	6 (8%)
	4 (%)	50 (17%)	8 (10%)
	5 (%)	18 (6%)	4 (5%)
Adverse events	Gallbladder perforation* (%)	86 (29%)	15 (19%)
	Major bile leakage* (%)	27 (9%)	1 (1%)
	Incidental finding* (%)	19 (6%)	3 (4%)
	Cholecystitis* (%)	55 (19%)	7 (9%)
	Without complication (%)	146 (50%)	56 (73%)

*Not mutually exclusive

### Deep learning model architecture

**Fig. 1:**
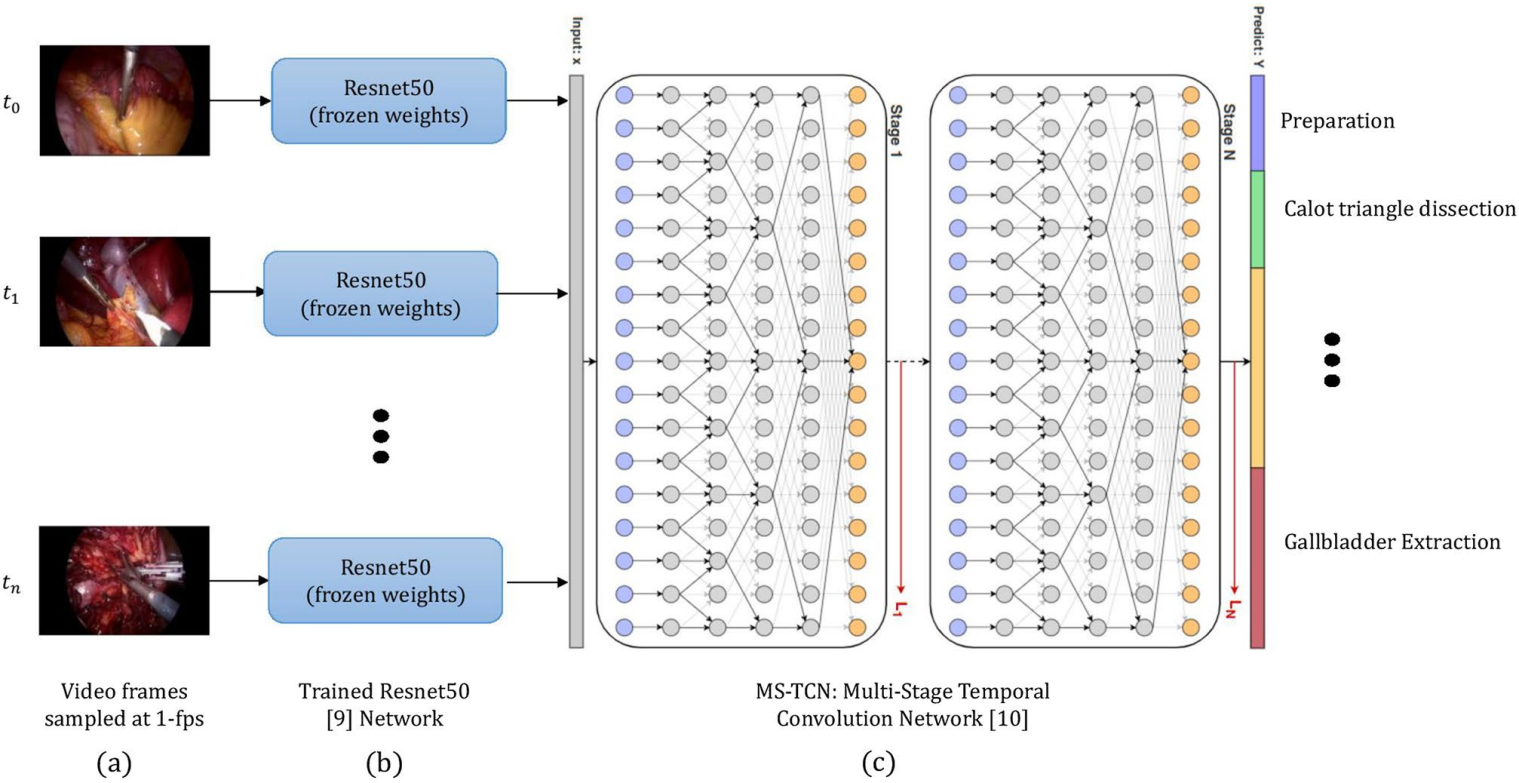
Overview of our proposed neural network, MS-TCN—Multi-Stage Temporal Convolution Network [22]. **a** The LC video is processed at 1 frame per second (fps). **b** Each frame is fed into a deep convolutional neural network—Resnet50 [9]. The Resnet50 model is trained to classify each frame’s associated surgical phase independently. Following training, the last prediction layer of the Resnet50 is removed, and all the network parameters are frozen (not trainable subsequently). For each frame, the Resnet50 produces a feature vector which expresses the visual information content of the frame as a lower dimensional (compared to the original frame) numerical “feature vector”. **c** All feature vectors from the input LC video are combined to form a sequence of feature vectors representing the entire LC video. This sequence is inserted to the MS-TCN model which consists of temporal convolution layers with a dilation rate that increases across layers. The temporal convolution layers capture temporal connections, and the increasing dilation setup enables the capturing of long term temporal dependencies. The final layer of the MS-TCN model outputs the surgical phase prediction for each frame in the video

Our model takes as input a video, and categorizes each frame in the video into 1 of the 10 phases described above. This is achieved using a two-stage setup (Fig. 1). The first stage extracts visual features from single frames of the video, without any temporal context, i.e., this stage of the model has no sense of what is happening before and after that frame. The second stage of the model aggregates temporal information from neighboring frames, i.e., it is at this stage that the model is able to incorporate information from both before and after that frame, to understand what surgical phase the current frame shows.

#### First stage: feature extraction model

Deep convolutional neural networks [17, 18] have recently shown state-of-the-art results on image classification tasks [19, 20]. While classical classification models focus on extracting hand-crafted features (colors, corners, edges, etc.), and combining them as inputs to supervised machine learning models, deep neural networks learn the features by themselves from the raw data. The extracted features are thus optimized to improve classification performance. In this work, we apply a deep residual convolutional neural network architecture called Resnet50 [21], to extract features from LC frames. Given a single frame taken from a cholecystectomy procedure as input, the goal of the Resnet50 model is to output its estimated likelihood of the frame being in each of the 10 phases. In other words, this model is trained to predict the cholecystectomy phases from single frames. When the training of the Resnet50 model is complete, the network weights are frozen (i.e., fixed for the remainder of the learning procedure), and the last prediction layer is removed. The resulting network is then able to output a single feature vector from each of the raw cholecystectomy frames.

#### Second stage: temporal aggregation model

While the Resnet50 model learns to identify the surgical phases based only on information from a single frame, our goal was to also incorporate the temporal patterns across LC videos. This is due to the fact that frames before and after a given frame are often helpful or even necessary to understand that frame. For example, the gallbladder dissection phase must precede other phases of the procedure. Thus, frames where the gallbladder appears, but which precede the gallbladder dissection phase, are unlikely to be part of the gallbladder packaging or gallbladder extraction phases.

For the temporal aggregation stage, we utilize a temporal convolution network [22, 23] which recently achieved state-of-the-art results on temporal action segmentation tasks in general videos [24, 25] as well as on surgical phase detection in the Cholec80 dataset [26]. (We note in passing that temporal information was also shown to be useful in analyzing suturing videos [27].) In our case we employ a variant of this network architecture, known as the Multi-Stage Temporal Convolution Network (MS-TCN) network [22] (Fig. 1). The MS-TCN network consists of multiple stages, where each stage is composed of so called “dilated temporal convolution blocks”. The purpose of these blocks is to efficiently aggregate information from the entirety of the procedure, allowing us to learn temporal dependencies over the whole cholecystectomy video. The network is multi-stage: each stage outputs an initial prediction that is refined by the next one. The input to the MS-TCN network is a sequence of feature vectors, one for each frame of video, as described in the previous section; the output is a phase prediction for each frame in the input sequence.

### Statistical analysis

We evaluated our model on the test set, using the accuracy metric. Accuracy quantifies the fraction of frames with correctly classified phases and is defined as the number correctly classified frames divided by the total number of evaluated frames. On average, a small fraction (0.16%) of the frames in each video was not annotated due to difficulties in selecting precise start/end frames for annotation in a way that eliminates unannotated gaps. The accuracy was calculated on both the first stage (Resnet50) model alone and the second stage (MS-TCN) model. This frame-level accuracy per-video was then averaged over all videos to ensure each video was equally weighted. For error bars, we computed the 95% empirical confidence interval (CI) by bootstrapping across videos. To place the model’s accuracy in perspective, each video was annotated by a second surgeon (as mentioned in the Annotation section). The inter-surgeon agreement was then computed by evaluating the second surgeon’s accuracy per-video against the first, and similarly averaging across all videos.

## Results

The first stage (Resnet50) model achieved overall classification accuracy of 78% [95% CI 75.8%, 80.1%] on the test set. The second stage (MS-TCN) model, which incorporates temporal information across the whole video, obtained higher accuracy, reaching 89% [95% CI 87.1%, 90.6%] accuracy on the test set.

**Fig. 2:**
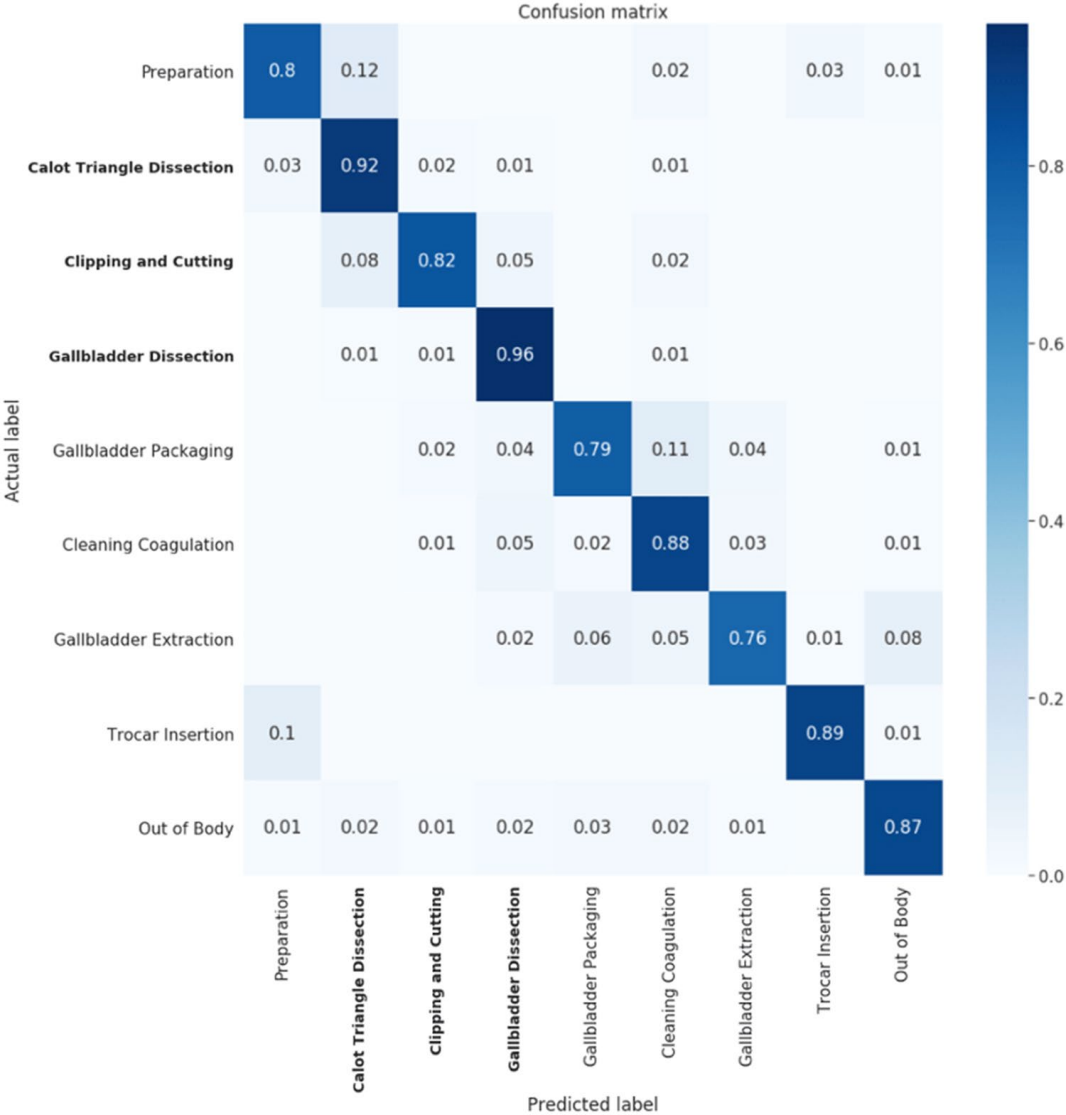
Normalized confusion matrix showing the accuracy achieved separately for each surgical phase. The critical phases in an LC procedure are in bold; 92% of the Calot triangle dissection phase frames were correctly classified and 96% of the gallbladder dissection frames were correctly classified. (Please note that the rows of the confusion matrix do not precisely sum to 1 due to rounding.)

Figure 2 shows evaluation of the per-phase confusion matrix, reached by the full 2-stage model. The per-phase calculation was performed across all frames per-video, and then averaged over all videos in the test set. We noted that the model successfully detected the most critical phases—Calot triangle dissection, clipping and cutting, and gallbladder dissection phase—with accuracies of 92%, 82%, and 96%, respectively. For the preparation phase, the model reached 80% accuracy; however, 12% of these preparation frames were incorrectly predicted as part of the Calot triangle dissection phase instead. We note that these erroneous predictions are distributed along the transition between the two phases.

**Fig. 3:**
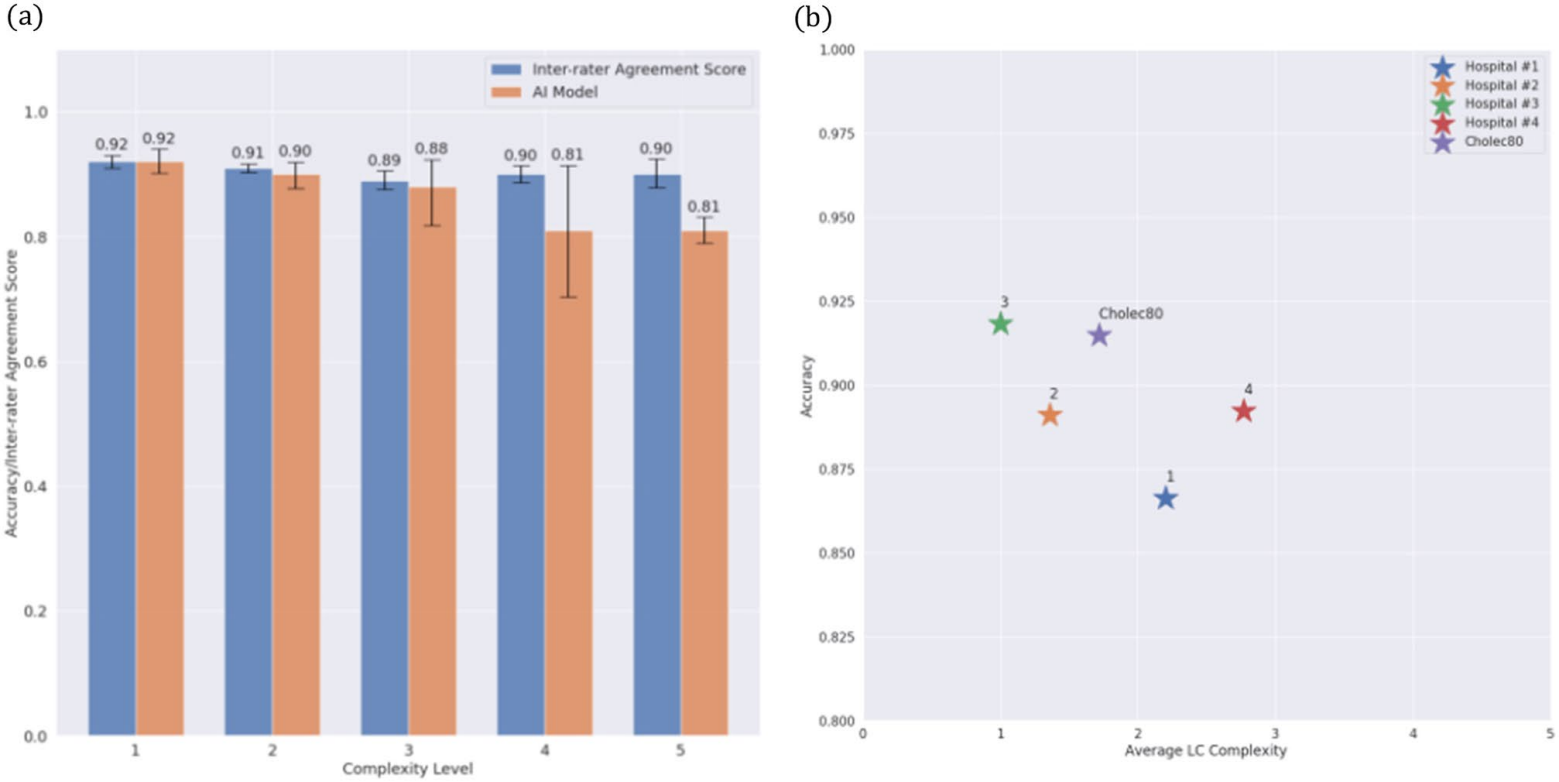
Accuracy of the MS-TCN and the inter-rater agreement score stratified by LC complexity level. **a** For the simplest LC procedures, the MS-TCN reaches 92% accuracy, similar to the agreement score between the annotators. As expected, as the complexity level increases, the accuracy of the MS-TCN model decreases. Nevertheless, for the most complex LC procedures (levels 4 and 5), the MS-TCN model reaches 81% accuracy. **b** Results stratified by institution; each marker in the graph represents a different hospital source. The x-value of each marker is the average complexity level of LC procedures for the given source hospital. The y-value of each marker is the average accuracy achieved by the AI model on LC procedures for the given source hospital

As described in the Annotation section, the complexity level of each LC video in the dataset was annotated on a scale of 1–5. Figure 3A shows the mean accuracy of our MS-TCN model (orange bars), and the inter-rater score agreement (blue bars) on the test set videos relative to their complexity. As the complexity increases from 1 to 3, the model’s accuracy linearly decreases from 92% [95% CI 90.2%, 94.0%] to 88% [95% CI 81.8%, 92.3%]. At complexity levels 4 and 5, the model accuracy was 81% [95% CI 78.9%, 83.1%].

The inter-rater agreement (between expert surgeons) ranged from 92% on LC procedures with a complexity level of 1 to 90% on LC procedures with a complexity level of 5. We learn from Fig. 3A that for simple LC procedures, the AI model has an ability equal to a surgeon in the recognition of surgical phases. However, on complex LC procedures, the surgeons are superior to the AI model: the annotator agreement score is 9% higher compared to the accuracy of the AI model.

**Fig. 4:**
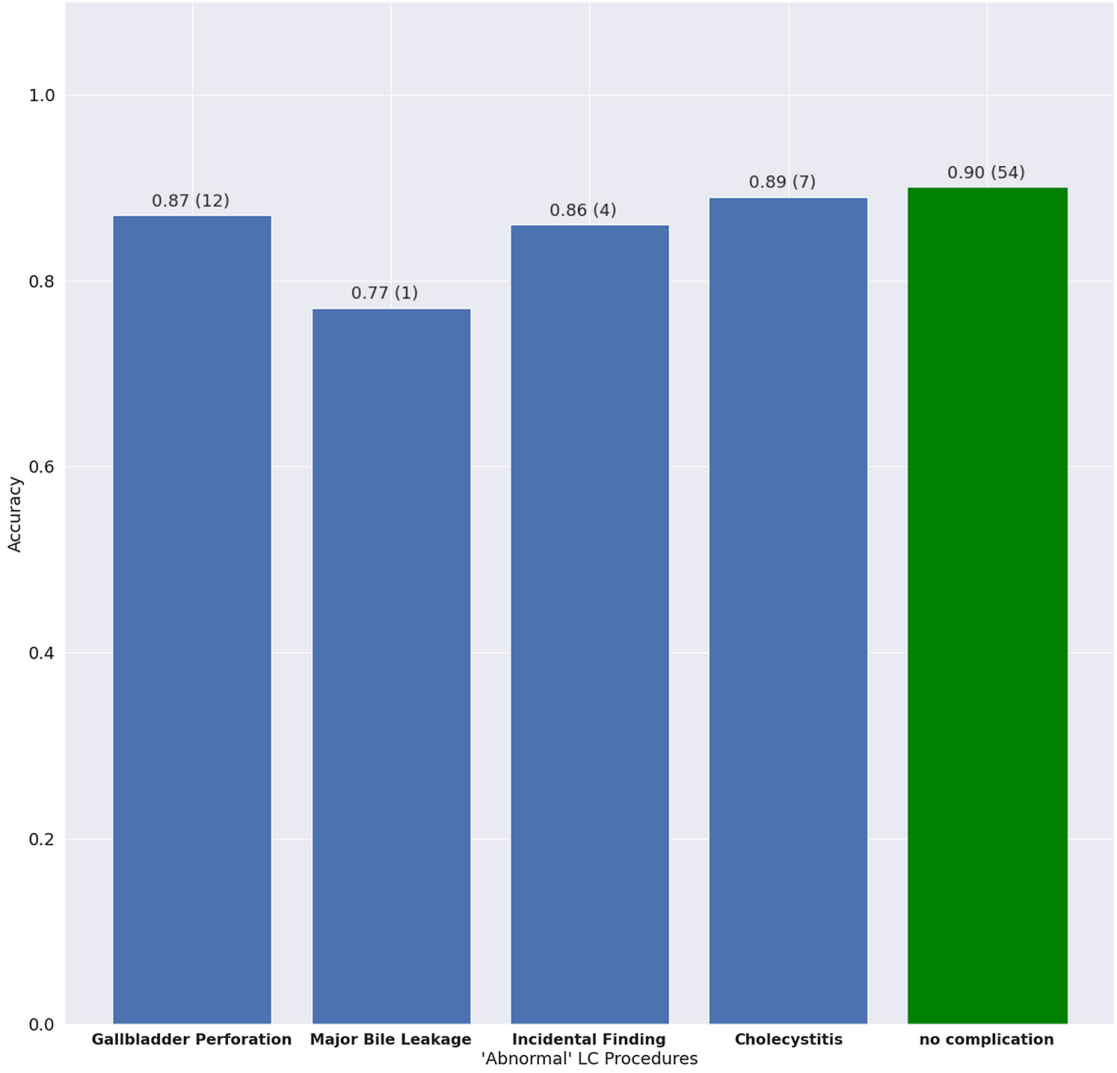
AI model accuracy stratified by presence of adverse events. Blue bars represent LC procedures where at least one adverse event occurred. The green bar represents regular LC procedures without occurrence of any adverse event. It can be seen that the AI model is robust to LC procedures with adverse events, reaching almost the same accuracy as in regular LC procedures. We note that only one procedure in the dataset contained a major bile leakage event

We evaluated how adverse events during LC procedures affect the ability of the AI model to recognize the surgical phases. Figure 4 shows the overall accuracy of the MS-TCN model on the test set, relative to adverse events in LC procedures. The model reached an accuracy of 87% [95% CI 82.5%, 90.7%] in videos with a gallbladder perforation event, 77% on a single video with a major bile leakage event, 86% [95% CI 76.8%, 94.1%] on videos with an incidental finding, and 89% [95% CI 88.7%, 92.6%] on procedures with cholecystitis (blue bars). On videos without adverse events (green bar), the model reached a mean accuracy of 90% [95% CI 88.0%, 91.7%]. Thus as expected, in LC procedures with adverse events, the AI model attained a lower accuracy.

The last research question we wanted to address was how LC procedures from different hospitals affect the ability of an AI model to recognize surgical phases. As described in the Dataset section, our dataset was composed of procedures from five hospitals. As may be expected, some variation was noted in the instruments used as well as in surgical technique. This made the task of identifying the surgical phases more challenging.

Figure 3B shows the overall accuracy of the MS-TCN model, according to both the source hospital as well as the average complexity level. The model attained an accuracy of 86% [95% CI 83.1%, 88.7%] in videos from hospital #1, 89% [95% CI 83.8%, 93.1%] on videos from hospital #2, 91.5% [95% CI 88.3%, 94.3%] on videos from hospital #3, and 89% [95% CI 84.8%, 93%] on videos from hospital #4. On videos from the Cholec80 [1] dataset our model reached an accuracy of 91.4% [95% CI 88.4%, 93.7%].

To understand how effectively the AI model generalizes to various hospitals, we trained the AI model on four of the hospitals and tested it on the fifth. We repeated this experiment five times, where each time a different hospital was set aside as the test set (with the remaining four used as the train set). eFigure 2 in the Supplement shows the average accuracy of the MS-TCN for each experiment. The model attained an accuracy of 79% [95% CI 72.4%, 84.9%] in videos from hospital #1, 84% [95% CI 81.3%, 87.3%] on videos from hospital #2, 89% [95% CI 86.0%, 90.6%] on videos from hospital #3, and 87% [95% CI 76.4%, 94.3%] on videos from hospital #4. Using the four hospitals to train the AI model, and testing it on the Cholec80 [1] dataset, the AI model reached an accuracy of 87% [95% CI 84.0%, 89.8%].

## Discussion

In our study, we have presented an AI model to automate the task of phase recognition in LC. Our model successfully detected surgical phases with an overall accuracy of 89%, comparable to the average agreement between surgeon annotators (90%), including successful detection even in procedures with adverse events like major bleeding, major bile leakage, major duct injury, and gallbladder perforation.

The detection of surgical phases is more critical for certain phases than it is for others. For example, successful identification of the Calot triangle dissection phase, confirmation of the critical view of safety (CVS), or the clipping and cutting phase, are of utmost importance for the patient’s safety, while misrecognition of the gallbladder extraction phase is less important and will have a much lower impact on patient safety. As shown, our system was able to reach a very high accuracy (92%) in the Calot Triangle Dissection phase that supports CVS.

We also found that higher complexity levels of LC procedures were associated with both lower accuracy on the part of the AI model, as well as lower inter-rater agreement between surgeons. On less complex LC videos, the AI model achieved an overall accuracy of 92%, equal to the inter-surgeon agreement score. By contrast, in complex LC videos, the annotators reached an average agreement score of 90% compared to 81% by the AI. Importantly however, the accurate identification of the Calot triangle dissection phase was unaffected in complex videos (92%). Furthermore, the performance of the AI model remained high in the presence of adverse events, indicating an overall robustness to adverse events during LC procedures. As mentioned, we used LC videos from five hospitals, and as expected, some variation in the surgical technique and type of instruments used was noted. Interestingly, such variations did not interfere with the accuracy of the AI system in phase recognition reaching 80–87% overall accuracy reflecting the system’s flexibility and reliability.

Non-realtime use of such an AI system to analyze LC videos may provide valuable data to evaluate and track trainees’ surgical skill level over time, and even enable future studies into correlations between specific events occurring during a procedure and outcomes such as successful conclusion of the procedure. Similarly, the AI system can enable finer-grained analysis of time taken for procedures, potentially providing insights that can augment systems which predict surgical duration and hence aid OR planning [28]. Further sophistication and modifications to enable real-time incorporation of such a system into the laparoscopic video camera system is possible, by using no future information, or limited future information (e.g., using a few frames of future information is unlikely to cause appreciable latency). Such real-time use may play a role in active monitoring to improve patient safety, by providing the surgeon with indications of the successful conclusion of the various surgical phases and alerting if there might be potential issues with the surgical view or dissection plane. For instance, if the system was not able to satisfactorily recognize the CVS, an alert could be generated to prompt re-evaluation of their perception of the anatomy, before proceeding to the clipping and cutting phase (which is irreversible). Although the overall complication rate and bile duct injury in LC is very low [29], such a system may improve safety in teaching departments where junior staff are undergoing training. Likewise, similar systems could aid real-time decision making such as whether to proceed with laparoscopy, to change the surgical technique (i.e., retrograde dissection or subtotal cholecystectomy), to convert to open surgery, to drain only, or to abort the procedure. Extensions of such a system to other more complex laparoscopic procedures, such as solid organs surgery, may also be useful.

AI has recently gained popularity in several medical fields like radiology, pathology, and gastroenterology [30–33]. However, unlike images of diagnostic radiology, the image quality of frames in surgical videos has a significantly greater variability owing to movement during video capture, which renders AI analysis more challenging. In addition, anatomical structures and surgical planes are often hidden under fatty tissue and must be exposed before yielding a clear field of view for an AI system’s interpretation. Previous studies on AI for interpreting laparoscopic videos [12, 17, 34–36] have focused on identifying procedure phases and instruments. In a recent study using a large data set of 1243 LC videos [13], the authors showed that AI performance was significantly improved when the number of videos of the input dataset was increased from 50 to 745. Compared to prior work, our dataset made crucial use of videos representing real-world variability across anatomy, surgeon’s technique, operative tools, surgical complexity, and intraoperative complications. In particular, our study included often-encountered complex procedures such as those requiring retrograde dissection, conversion to open procedure, and cholecystitis of varying severity.

This study has several limitations. The AI model was trained to recognize only the normal surgical phases (preparation, Calot triangle dissection, etc.) in videos which included adverse events. However, during an adverse event, the scene might not be related to the current surgical phase, which may have impacted the AI model’s performance in the presence of adverse events. Future work into adapting the AI model to additionally recognize adverse events may help improve performance. In a related vein, some adverse events were rare. For example, only one LC procedure contained a major bile leakage, so additional examples of rarer adverse events would be helpful for both training and evaluating the model to correctly identify such events. Another limitation relates to the non-real time nature of the system, which does not allow it to be used to provide safety indications during the procedure. As noted above, future work will focus on training the network in such a way as to accommodate real-time operation.

In conclusion, this study presents an AI system for accurate recognition of predefined surgical phases in both uncomplicated LC procedures and complex procedures. This study is a first step toward further development of an AI system for surgical skill assessment, efficient OR schedule planning, and importantly to assist the surgeon in avoiding technical errors, alert them to imminent complications, and provide real-time information to be used for better decision making.

## Supplementary Information

Below is the link to the electronic supplementary material.

Supplementary file1 (DOCX 163 KB)
