# Supplementary material for: Artificial intelligence for phase recognition in complex laparoscopic cholecystectomy
Source: Surg Endosc. 2022 Aug 8;36(12):9215–23. doi: 10.1007/s00464-022-09405-5 (PMC9652206; doi:10.1007/s00464-022-09405-5)
Supplement: Supplementary file 1 — Supplementary file1 (DOCX 163 KB) [file 464_2022_9405_MOESM1_ESM.docx]

Artificial Intelligence for Phase Recognition in Complex Laparoscopic Cholecystectomy - Supplementary Material

## Deep Learning Model Architecture

### Neural network architecture and training

We used the Multi-Stage Temporal Convolution Network (MS-TCN) [1] as the architecture. This architecture allows the model to capture long-range time dependencies and recognize temporal phase segments. We use a MS-TCN architecture with five stages, with each stage containing 19 dilated convolution layers, where the dilation factor is doubled at each layer and dropout is used after each layer. Similar to [1], all layers had 64 filters, each of size 3 and a ReLU (rectified linear) activation. We further use residual connections to facilitate gradient flow. To get the probabilities for the output phase for each frame, we apply a 1 × 1 convolution over the output of the last dilated convolution layer followed by a softmax activation. We use the Adam optimizer with a learning rate of 0.0001 and minimize the average cross-entropy loss. The training runs for 50 epochs with the final model being the one with the best validation results during the optimization process. Batch size is a tuned hyperparameter (described next), and all videos were resampled to 1 frame/second, and zero-padded to the longest video in each batch.

### Hyperparameter tuning

The hyperparameters were chosen among the following options: number of dilated layers in each stage (5, 7, 10, 12, 15, 17, 19, 20), number of stages (1, 2, 3, 4, 5), batch size

(4, 8, 16, 32), learning rate (0.01, 0.001, 0.0001), optimization algorithms

(SGD, RMS, ADAM), dropout rate (0, 0.5, 0.8). In addition, we also attempted adding an additional smoothing loss [1] and using different feature extractor models (Inception V3, I3D).

## Annotation of Surgical Phases, Adverse Events, and Level of Surgical Complexity

### Adverse Events and Surgical Complexity Annotations

The surgeons were asked to grade the complexity level of each surgery in a scale of 1-5 based on intraoperative parameters. The factors to determine the complexity level included: state of the gallbladder (based on the Parkland Grading Scale for grading still images of Cholecystitis [2], [3]), presence of intra-abdominal adhesions, normality of anatomy, duct closure device utilized, performance of intraoperative cholangiography, partial or open cholecystectomy requirements and intraoperative adverse events.

| Complexity Grade | Description |
| --- | --- |
| 1 | - **State of gallbladder***: Normal appearing gallbladder - No adhesions present - Completely normal gallbladder - Normal anatomy. - No presence of intra-abdominal adhesions. - Duct closure device utilized with **clip**. - No performance of cholangiography. - No requirements for partial or open cholecystectomy. - No intraoperative adverse events. |
| 2 | - **State of gallbladder*:** Minor adhesions at gallbladder neck, otherwise normal gallbladder - Adhesions restricted to the neck or lower of the gallbladder - Normal anatomy. - Minor presence of intra-abdominal adhesions. - Presence of a single minor bile-leakage event which takes less than 1 minute. Duct closure device utilized with clip. - No performance of cholangiography. - No requirements for partial or open cholecystectomy. |
| 3 | - Presence of **ANY** of the following: - **State of gallbladder**: - One of, hyperemia, pericholecystic fluid, adhesions to the body, distended gallbladder - Medium presence of intra-abdominal adhesions. - Duct closure device utilized with strip - Performance of cholangiography - Multiple events of gallbladder perforation/bile leakage that takes more than 1 minute each. - No requirements for partial or open cholecystectomy. - Normal anatomy. |
| 4 | - Presence of **ANY** of the following:   - **State of gallbladder***:     - adhesions obscuring majority of gallbladder     - Intrahepatic gallbladder.     - impacted stone (Mimzi)   - Abnormal liver anatomy   - Adverse events. One of:     - Major bile leakage     - Major gallbladder perforation     - Major bleeding handled with suction irrigator. - No requirements for partial or open cholecystectomy. |
| 5 | - Presence of **ANY** of the following:   - **State of gallbladder***:     - Perforation, necrosis, inability to visualize the gallbladder due to adhesions.   - Adverse events. One of:     - Major bleeding handled with gauze pads     - Bile duct injury     - Bleeding of the liver     - Bile leakage from the liver itself     - Ulceration of the liver     - Tumor identification (rare)   - Move to partial or open cholecystectomy   - Multiple difficult anatomies. |

**Table 1. Cholecystectomy complexity grading scale.**

(*) Grade of gallbladder state was based on the Parkland grading scale for cholecystitis [2], [3].

| Complexity level | Hospital #1 | Hospital #2 | Hospital #3 | Hospital #4 | Cholec80 | Total |
| --- | --- | --- | --- | --- | --- | --- |
| 1 | 5 | 4 | 3 | 2 | 7 | 21 |
| 2 | 17 | 3 | 0 | 2 | 16 | 38 |
| 3 | 5 | 0 | 0 | 0 | 1 | 6 |
| 4 | 5 | 0 | 0 | 3 | 0 | 8 |
| 5 | 3 | 0 | 0 | 1 | 0 | 4 |
| Total | 35 | 7 | 3 | 8 | 24 | 77 |

**Table 2. Breakdown of complexity level of each procedure within the test-set, per hospital institution.**


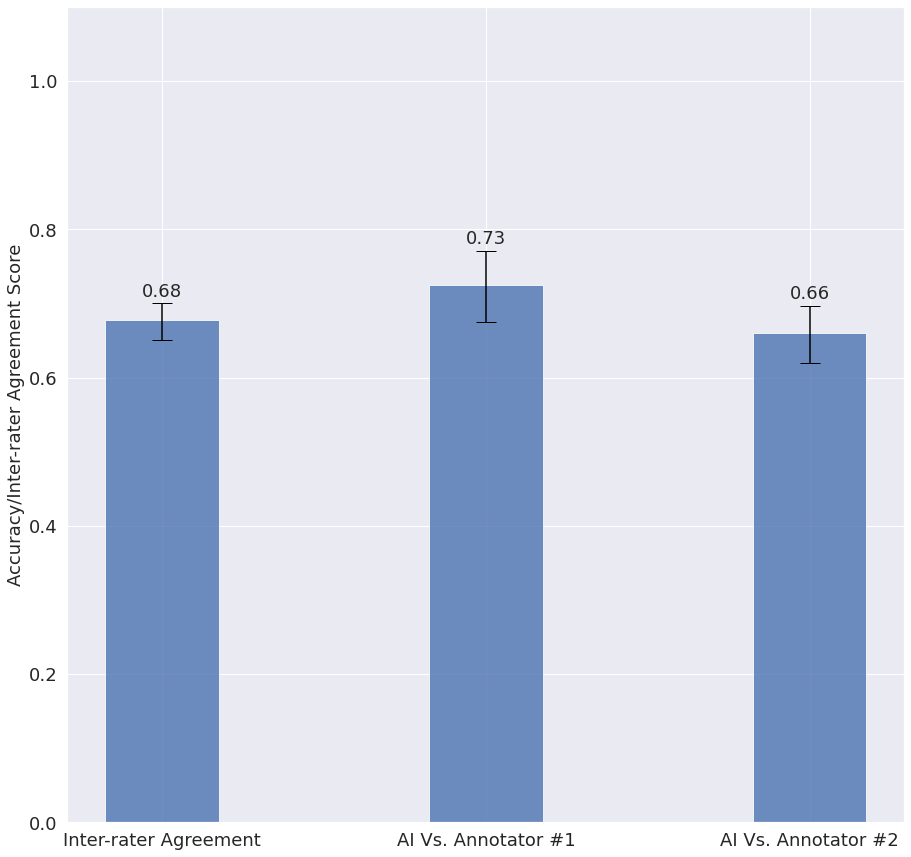


**Figure 1. Analysis on videos which were excluded from the dataset due to interrater agreement below 80%.**


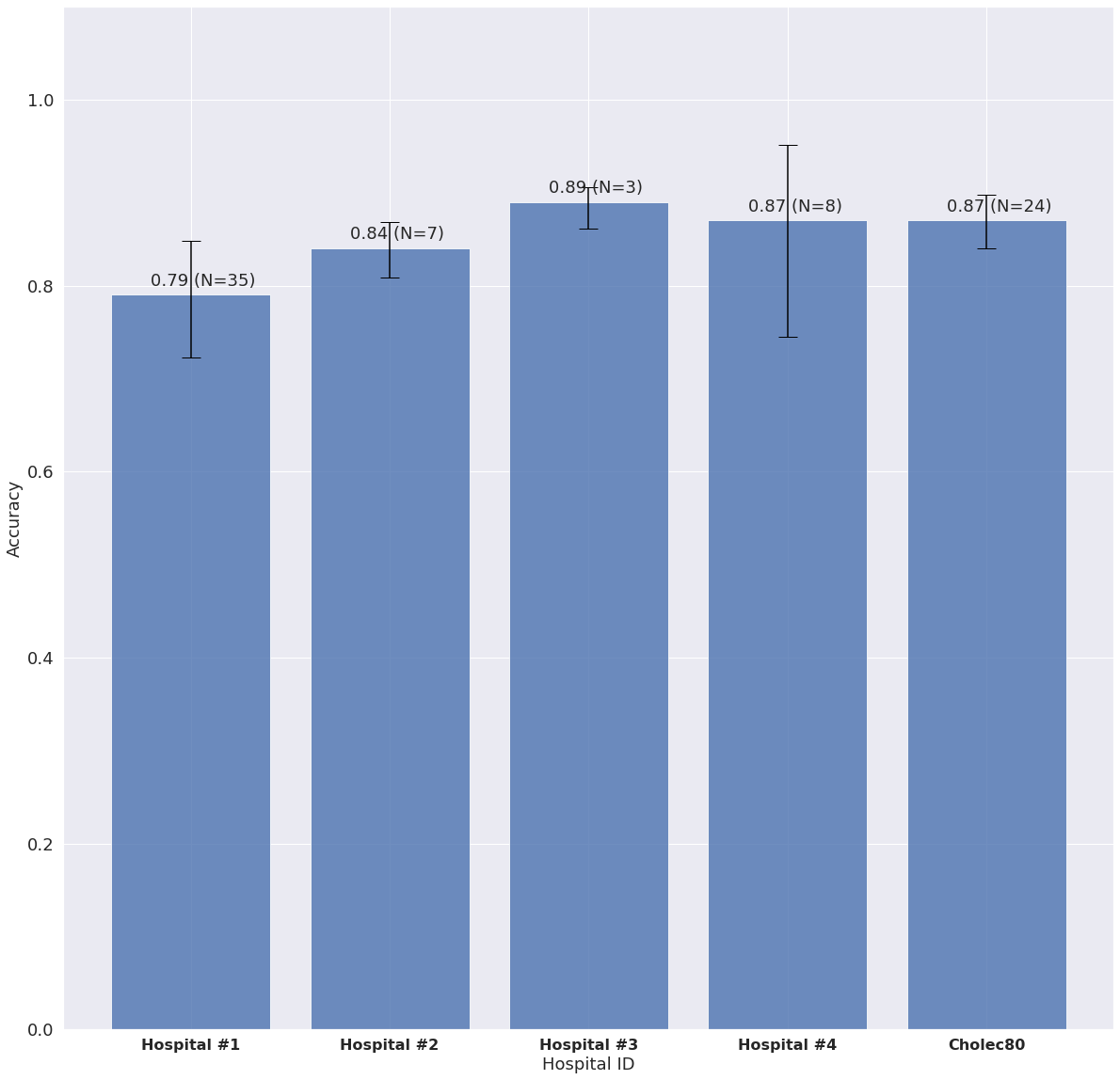


**Figure 2.** Each bar shows the accuracy reached by the AI model on an external hospital, when the other four were used to train the model.

# **REFERENCES**

[1] Farha YA, Gall J. MS-TCN: Multi-Stage Temporal Convolutional Network for Action Segmentation. 2019 IEEE/CVF Conference on Computer Vision and Pattern Recognition (CVPR). Epub ahead of print 2019. DOI: 10.1109/cvpr.2019.00369.

[2] Madni TD, Leshikar DE, Minshall CT, et al. The Parkland grading scale for cholecystitis. Am J Surg. 2018;215:625–630.

[3] Madni TD, Nakonezny PA, Barrios E, et al. Prospective validation of the Parkland Grading Scale for Cholecystitis. *Am J Surg*. 2019;217:90–97.
